# Parental and health professional evaluations of a support service for parents of excessively crying infants

**DOI:** 10.1186/s12913-019-4430-5

**Published:** 2019-08-22

**Authors:** Deborah Bamber, Charlotte Powell, Jaqui Long, Rosie Garratt, Jayne Brown, Sally Rudge, Tom Morris, Nishal Bhupendra Jaicim, Rachel Plachcinski, Sue Dyson, Elaine M. Boyle, Nicole Turney, Joanne Chessman, Ian St. James-Roberts

**Affiliations:** 1 Nursing and Midwifery Research Centre, De Montfort University, Leicester, UK; 2 Counseling Psychologist and CBT Practitioner, Leicester, UK; 3 Leicester Clinical Trials Unit, University of Leicester, Leicester, UK; 4 National Childbirth Trust, London, UK; 5 School of Health and Education, Middlesex University, London, UK; 6 Department of Health Sciences, University of Leicester, Leicester, UK; 7 Leicestershire Partnership NHS Trust, Leicester, UK; 8 Thomas Coram Research Unit, UCL Institute of Education, University College London, London, WC1H OAL UK

**Keywords:** Infant crying, Parenting, Healthcare, Health services

## Abstract

**Background:**

The ‘Surviving Crying’ study was designed to develop and provisionally evaluate a support service for parents of excessively crying babies, including its suitability for use in the United Kingdom (UK) National Health Service (NHS). The resulting service includes three materials: a website, a printed booklet, and a Cognitive Behaviour Therapy (CBT) programme delivered to parents by a qualified professional. This study aimed to measure whether parents used the materials and to obtain parents’ and NHS professionals’ evaluations of whether they are fit for purpose. Parents were asked about participating in a randomised controlled trial (RCT) to evaluate the materials fully in health service use.

**Methods:**

Participants were 57 parents with babies they judged to be crying excessively and 96 NHS Health Visitors (HVs). Parental use and parents’ and HVs’ ratings of the Surviving Crying materials were measured.

**Results:**

Thirty four parents reported using the website, 24 the printed booklet and 24 the CBT sessions. Parents mostly accessed the website on mobile phones or tablets and use was substantial. All the parents and almost all HVs who provided data judged the materials to be helpful for parents and suitable for NHS use. If offered a waiting list control group, 85% of parents said they would have been willing to take part in a full RCT evaluation of the Surviving Crying package.

**Discussion and conclusions:**

The findings identify the need for materials to support parents of excessively crying babies within national health services in the UK. The Surviving Crying support package appears suitable for this purpose and a full community-level RCT of the package is feasible and likely to be worthwhile. Limitations to the study and barriers to delivery of the services were identified, indicating improvements needed in future research.

**Trial registration:**

Study Registration no. ISRCTN84975637.

## Background

During the first four postnatal months, some infants cry for prolonged periods without an apparent reason. The prevalence is uncertain, but estimated to be around 20% [1, 2]. Until recently, the main focus for research has been on the crying and its causes [3–5]. However, it is becoming clear that an equal focus on parents and subsequent outcomes is needed. One reason for that need is that no consistently effective treatment for the infant crying has emerged [5]. Another is that parental concerns generate health service use and costs, which are substantial [6]. There is evidence, too, that crying judged by parents to be excessive can trigger premature termination of breastfeeding [7], over-feeding [8], parental distress and depression [9, 10], poor parent-child relationships [11], and infant abuse [12].

As well as its loud and aversive sound, many parents find crying bouts which resist soothing manoeuvres - a feature specific to the first four postnatal months - to be frustrating [13]. However, the effect on parental emotions and actions depends partly on how parents cope with the crying, which involves parental resources, vulnerabilities and circumstances. Factors such as depression, anxiety and high arousal influence how parents interpret and respond to infant crying [14, 15]. Social isolation, although less studied, may also increase its adverse consequences. Parental vulnerabilities have been found to increase the likelihood of serious long-term child disturbances [16].

Based on this evidence, studies have begun to develop materials which support parents who judge their baby’s crying to be excessive [17–20]. Some seek to prevent infant abuse, but others have the broad goal of helping parents to manage infant crying and their responses to it.

The ‘Surviving Crying’ study was designed to develop and provisionally evaluate a package of support materials for United Kingdom (UK) parents of excessively crying babies, including whether the materials might be suitable for use in the National Health Service (NHS). It was intended to explore whether a full-scale randomised controlled trial was feasible and worthwhile and to develop the materials and methods for such a trial. The study included academic, NHS and voluntary sector partners. In particular, specialist community nurse Health Visitors (HVs) and their teams, who provide health services for all UK parents with infants, were closely involved. The resulting materials, comprising a website, a printed booklet, and a programme of Cognitive Behaviour Therapy (CBT)- based support sessions delivered to parents by a qualified practitioner, have been described [21, 22] and provisional evidence that they are associated with reductions in parental frustration, anxiety and depression has been reported [23]. Since their use in the NHS depends on whether parents and NHS professionals find them suitable, this study aimed to measure parents’ use of the materials and parental and HV evaluations of whether they are fit for purpose. Parents were also asked about their willingness to participate in a full randomised controlled trial (RCT) of the materials.

The phrase ‘excessive infant crying’ is used here to refer to a parent’s judgment that an infant is crying too much, often accompanied by a concern that the crying signifies the infant is unwell. The phrase ‘prolonged infant crying’ refers to an objective measure of crying duration. The abbreviation ‘HV’ is used to include Health Visitors and allied NHS professionals who work with them to deliver health visiting services.

## Methods

### Governance

Study public registration no. ISRCTN84975637; ethical approval provided by De Montfort University (13450) and the National Research Ethics Committee East Midlands (Nottingham) (project ID 152836; NRES: 14/EM/1202).

### Recruitment of participants

Recruitment involved collaboration with 12 HV centres in city, suburban and rural areas of one UK East Midlands NHS Trust. HVs were introduced to the study, definition of ‘excessive crying’ and materials at briefing workshops and gave written informed consent if they chose to take part. In total, 124 HVs consented to participate. They were invited to visit the study website and provided with log-on information.

Where a parent expressed concern about excessive infant crying to a participating HV, the HV gave the parents brief written details about the study and sought consent for contact details to be passed to the research team. The research team then contacted parents to explain the study fully, confirm eligibility and invite them to complete a consent form. Inclusion criteria were (1) a parent of a healthy first or later-born infant aged ≤6 months judged by the parent to be crying excessively; (2) English speaking or supported by an English speaker; (3) living within the study area. Parents who did not meet these criteria were excluded. Because this study aimed to provide an initial evaluation of the Surviving Crying materials there was no control group.

### Assessments

Baseline assessments, including demographic measures, were conducted after recruitment through face-to-face interviews. At the interview, parents were asked to choose which elements of the support package they wished to receive. They could receive all three and as often as they chose. The information booklet was handed to participants at the end of the assessment, whilst parents who wished to access the study website were given a card containing its address and personalised log-in details. Parents who chose the CBT sessions were given contact details for the CBT Practitioner and their contacts were passed to the Practitioner, who made contact if that had not already occurred.

It was envisaged that outcome interviews would be conducted 4–6 weeks after baseline assessments, allowing website and booklet access for ≥4 weeks and delivery of up to five CBT sessions. In practice the mean interval was five weeks five days (range one week six days to 13 weeks six days). Parental use of the materials was assessed by self-completed questionnaires; website use was also monitored automatically using Google Analytics sotware [24]. Parents’ evaluations of each package element were obtained using rating scales and free text comment boxes. Following an explanation of the randomisation and control group processes involved in RCTs, parents were asked whether they would have been willing to participate in such a trial, with or without the option of a waiting list control group. The waiting list control group would be offered the Surviving Crying materials after a delay of one month.

All assessments occurred in parents’ homes and parents received a high street shopping voucher to acknowledge their contribution.

Following the close of recruitment and assessment of parents, the 124 HVs who gave consent to participate in the study were re-contacted and asked to complete a questionnaire to give their views on the package materials and their suitability for NHS use.

### Data analysis

Descriptive statistics were generated and free text comments were summarised to highlight key themes.

## Results

Parental recruitment lasted May–November 2016. Detailed recruitment and descriptive figures for participants are included in Powell et al. [23] In total, 57 parents provided data. The majority (94%) were mothers and married or co-habiting (92%). They were predominantly white, but small numbers of Asian, Black, Mixed and Other-ethnicity parents took part. Forty three percent had university degrees and a further 10% post-‘A’ level vocational qualifications. Many (69%) were on maternity or paternity leave at the time when their baby cried excessively. Five couples participated, giving information for 52 infants. Proportions of male and female infants were similar (47% male, 53% female). Just under half (44%) of the infants were first-born, 44% second born, 12% later born. The infants’ excessive crying began at a mean age of 3.1 weeks (SD 2.8 weeks). Their mean (SD) age at study entry (baseline) was 9.6 (5.6) weeks, range 3–24 weeks. At outcome assessment, their mean (SD) age was 15.3 (5.6) weeks, range 9–30 weeks [23]. Overall, 55 parents requested access to the website, 27 the printed booklet and 32 the CBT sessions (they could request all three elements). Outcome data were obtained for 52 parents; five could not be re-contacted.

### Parental use of the surviving crying materials

Of 52 parents who opted to receive the support package and provided outcome data, 49 (94%) reported using at least one of the three package materials (Table 1). The website was the most commonly used element, accessed by 30 individual parents (58%) with four of them accessing the website on both a mobile phone and computer/tablet.

**Table 1:** Parents’ use of the Surviving Crying materials

(a) Parental report figures^a^	
Total number of parents reporting use	52
Any materials accessed	49
Accessed website	34^b^
Read printed materials	24
Attended practitioner CBT sessions	24
(b) Google Analytics figures for website use	
Number of users	34
Number of sessions	54
Average length of session	6 min 1 s
Average number of unique page views per session	6.02
Average length of page view	1 min 27 s

^*a*^*Parents could use more than one package material.* ^b^Website accessed by 30 individual parents with four accessing it both on mobile phone and tablet/computer

The Google Analytics records confirmed that parents mostly used mobile phones or tablets to access the website (57 and 31% of sessions respectively); 11% used a desktop or laptop computer. As Table 1 shows, the 34 parents accessing the website had 54 viewing sessions between them: some visited the site more than once. Parents viewed a mean of six separate pages per session and spent 1 min 27 s on a page. However, some pages were viewed for much longer, e.g. the mean for the ‘Need Help Now’ page was 10.2 min and 9.2 min for a parent video describing their experience of excessive infant crying and life after it resolved. Others among the 10 longest-viewed pages were two other parent videos and pages providing information about infant crying and its management.

Twenty-four parents (46%) reported reading the printed booklet and 24 (46%) received at least one CBT support session (Table 1). All sessions were delivered one-to-one or to couples, with some mothers participating in both. Men attended only with partners. Although the option of group sessions was planned, too few parents were available in the same area at the same time for this to be possible. All sessions took place in parents’ homes.

In total, 40 CBT sessions were delivered. Parents received a mean of 2 sessions (ranging from 1 to 4). The mean session length was one hour six minutes (range 45 min to one hour 45 min). Non-participant family members – partners, or in one case other family members - were unexpectedly present at five sessions. Most sessions (93%) had a least one child, and 22% two or more children, in attendance.

### Parental evaluations of the surviving crying materials

Table 2 summarises parents’ ratings of the support materials’ usefulness. All 52 parents reported the materials they accessed to be ‘very useful’ or ‘useful’ and all 52 considered that the materials should be routinely included in the NHS. The parents’ free text comments noted that the materials offered reassurance and hope, practical help, advice and information.

**Table 2:** Parental ratings of the Surviving Crying materials’ usefulness and use in the NHS

*Usefulness: n (%)*	*Very useful*	*Useful*	*No Opinion*	*Not Useful*	*Total n*
Website	15 (44.1%)	19 (55.9%)	0 (0.0%)	0 (0.0%)	34
Printed booklet	16 (66.7%)	8 (33.3%)	0 (0.0%)	0 (0.0%)	24
Practitioner CBT sessions	20 (83.3%)	4 (16.7%)	0 (0.0%)	0 (0.0%)	24
*Materials should be routinely included in the NHS: n (%)*	*Strongly Agree*	*Agree*	*No Opinion*	*No*	*Total n*
Website	28 (82.3%)	6 (17.6%)	0 (0.0%)	0 (0.0%)	34
Printed booklet	17 (70.8%)	7 (29.2%)	0 (0.0%)	0 (0.0%)	24
Practitioner CBT sessions	20 (83.3%)	4 (16.7%)	0 (0.0%)	0 (0.0%)	24

### Parental willingness to take part in a randomised controlled trial (RCT)

Parents were asked whether, at the time when their baby was crying excessively, they would have been willing to take part in a RCT to provide a more rigorous evaluation of the Surviving Crying materials. The randomisation process and possibility that they would be assigned to the control group was explained, together with the option of a waiting list control group. Sixty nine percent of parents said they would have been willing to take part, rising to 85% if the option of a waiting list control group was offered.

### HV contact with and evaluation of the Surviving Crying materials

Table 3 summarises HV contact with the Surviving Crying materials. Of 124 who consented 96 (77%) provided feedback.

**Table 3:** HV contact with the study materials^a^

Total Number of HVs	96
Study website	
Seen directly: n contacts	29
Told about by parents: n contacts	4
Told about by colleagues: n contacts	25
Not seen or heard about the website: n (%) HVs	36 (37.5)
Study printed booklet	
Seen directly: n contacts	27
Told about by parents: n contacts	3
Told about by colleagues: n contacts	23
Not seen or heard about the booklet: n (%) of HVs	45 (47)
CBT session material	
Seen directly: n contacts	8
Told about by parents: n contacts	12
Told about by colleagues: n contacts	17
Not seen or heard about the CBT material: n (%) of HVs	54 (56)

^a^HVs could have had more than one type of contact with each material, so that numbers of contacts may not equal numbers of HVs

HVs reported most contact with the website either directly or by being informed about it by colleagues or by parents. They had the least contact with the CBT session materials. A high proportion of HVs reported not having seen the website, printed booklet or CBT session material (36, 45, and 54 HVs respectively) despite being introduced to the materials at the initial briefing sessions and being provided with website log on details. These limited contacts with the materials probably constrained the numbers of HVs able to complete the questionnaire section asking them to rate the materials. However, of the 51 (53%) HVs who rated at least one of the study materials, 95% rated them as ‘helpful’ or ‘very helpful’ (Table 4). Similarly, of 39 (41%) HVs who provided data on the materials’ suitability for inclusion in the NHS, over 85% rated them as ‘suitable’ or ‘very suitable‘.

**Table 4:** HV ratings of the Surviving Crying materials helpfulness and suitability for inclusion in the NHS

Helpfulness of material for parents (Total *n* = 51)	Very helpful	Helpful	Not helpful	n
Website n (%)	30 (69.8%)	11 (25.6%)	2 (4.7%)	43
Printed booklet n (%)	26 (61.9%)	14 (33.3%)	2 (4.8%)	42
Practitioner CBT sessions n (%)	28 (73.7%)	8 (21.1%)	2 (5.3%)	38
Materials should be routinely included in the NHS (Total *n* = 39)	Very suitable	Suitable	Not suitable	N
Website n (%)	23 (69.7%)	8 (24.2%)	2 (6.1%)	33
Printed booklet n (%)	20 (58.8%)	11 (32.4%)	3 (8.8%)	34
Practitioner CBT Sessions n (%)	17 (60.7%)	7 (25%)	4 (14.3%)	28

### Barriers to inclusion of the support materials in the NHS

HVs were asked to identify barriers to including the study materials in routine NHS services, and to suggest ways of overcoming these barriers. Over half (40 of 73) HVs who responded identified no barriers; 33 foresaw barriers to including the website, 28 the printed booklet and 26 the CBT materials in the NHS. The most common concern, identified by 27 (37%) of HVs, was language barriers, which would prevent use of the materials when parents neither spoke nor read English. Wider cultural, communication and literacy issues, as well as financial barriers, including not having internet access, were also cited**.** A further concern, raised by seven (10%) HVs, was that the amount of information they already need to communicate to parents within their visits would make it difficult to introduce additional information and services.

### Training for HVs in supporting parents with excessively crying babies

Of the 96 HVs, 90 (94%) agreed that supporting parents of excessively crying babies should be included in their training, just four disagreed and two were unsure. Of the 90 who agreed, 58 considered this should be part of routine HV training, and 40 that it should be part of specialist training, with some proposing both were needed.

## Discussion

The ‘Surviving Crying’ materials were designed to provide information, support and guidance for United Kingdom (UK) parents who are concerned about their baby’s excessive crying. This exploratory study aimed to measure parents’ use of the materials and to obtain both parents’ and health service professionals’ evaluations of whether they are fit for purpose. With a view to a future large scale evaluation, parents were also asked about their willingness to participate in a randomised controlled trial (RCT).

With some provisos, the findings are encouraging and indicate that further development and a full trial of the materials is worthwhile. Most parents used at least one of the support materials, i.e. the website, printed booklet and/or CBT-based support sessions. The website was accessed most often, mainly via mobile phone or tablet, and Google Analytic records confirmed that website use was substantial. All 52 parents and 95% of the community nurse Health Visitors (HVs) who provided data rated all three support materials as useful for parents. All 52 parents and most (≥85%) HVs giving an opinion judged that the materials should be included in the National Health Service (NHS).

The provisos to these findings involve limitations of the study which provide lessons about improvements needed in a future trial. The first is that our assumption that non-English-speaking parents would be supported in accessing the support service by English speaking family and friends proved to be incorrect. Particularly in light of the ethnic diversity in the study area, numbers of Asian, Black, Mixed and Other-ethnicity parents were small, indicating that a significant proportion of parents were not able to access the materials or to provide culturally-specific feedback on them. Ways of overcoming language, cultural and literacy barriers to delivery of the support materials, including by using more pictorial elements, are needed.

Second, although we planned to include small groups in the Cognitive Behaviour Therapy (CBT) part of the study, too few parents were available in the same area at the same time, so that all the CBT sessions were with individual parents or couples. Improvements, including the use of online discussion boards and social media, should be examined in future research.

Third, although two thirds of HVs had seen or heard about one or more of the Surviving Crying materials, only a third had seen the website and/or the printed booklet and only 8% the CBT materials. Probably as a result, only a half (51%) of HVs provided evaluation data. This study took place while HV services were being restructured and this, together with busy HV schedules, may have contributed to these low numbers. Although the responses of these professionals were highly positive, care is needed in generalising this finding to all HVs, while future studies may seek to improve HV involvement and how it is monitored and measured.

A final proviso is that some HVs expressed concern about their capacity to deliver the Surviving Crying materials.

Currently, UK HVs provide universal contacts for all parents with young babies at 10–14 days and 6–8 weeks after childbirth, usually via home visits, and are the obvious NHS professionals for delivering the proposed supports. In this NHS area, up to two further ‘universal plus’ home visits, or four clinic contacts, can be provided where there is additional need. In principle, this service provision could allow the website or printed materials to be introduced to all parents within the universal contacts, with the CBT programme offered as a universal plus service. Our finding that parents took up 1–4 CBT sessions each lasting approximately one hour fits this service model well.

In practice, although the Surviving Crying materials could enhance HV evidence-based services, these are under pressure and the amount of information HVs already need to communicate to parents makes it difficult to add anything new. Delivery of the CBT programme also requires special training. We note that 94% of HVs providing data considered that supporting parents of excessively crying babies should be included in their training, while the sustained collaboration in the research of a NHS Trust and over 90 of its staff highlight that these professionals value the support package and recognise the need for it. However, how to deliver the Surviving Crying materials, and how to pay for these new services, are questions which need to be tackled alongside further research. These questions are, to some extent, specific to the UK NHS, but how to embed provisions of this sort within healthcare services are generic issues which other countries and services may wish to consider.

## Conclusions

In conclusion, this study has found that there is an unmet need for services to support parents of excessively crying babies in the UK NHS and that parents, and many HVs, consider the materials examined here to be suitable for this purpose. As well as full-scale trials to establish whether the findings can be replicated at a community level, discussions with the professions, policy makers and commissioners involved are required if the promise contained in the findings is to be fulfilled.

## Data Availability

The data and access to the Surviving Crying materials are available on request from the Corresponding Author.

## Abbreviations

CBT: Cognitive Behaviour Therapy
HV: The abbreviation ‘HV’ is used to include Health Visitors and allied NHS professionals who work with them to deliver health visiting services
NHS: National Health Service
RCT: Randomised Controlled Trial
UK: United Kingdom
